# Medical Doctors’ Perceptions of the Media Coverage during the Covid-19 Pandemic: A Case Study in Stockholm

**DOI:** 10.1177/11786329231222168

**Published:** 2023-12-25

**Authors:** Clara Brune, Janne Agerholm, Ann Liljas

**Affiliations:** Karolinska Institute, Stockholm, Sweden

**Keywords:** Emergency medicine, Covid-19, media coverage, Stockholm, emergency department, media report

## Abstract

The strain on healthcare systems including emergency departments increased substantially during the Covid-19 pandemic,negatively affecting healthcare workers and their well-being. The emotional distress experienced by healthcare staff during the pandemic was worsened by confusion and conspiracy theories that circulated in the news and online media. Reports on the pandemic and general consumption of media intensified as the public’s demand for information increased. There is limited research on how doctors perceived media coverage, and how they were affected in their work. This study aimed to explore how medical doctors in emergency departments perceived the media coverage during the Covid-19 pandemic. Twelve doctors at two different emergency departments in Stockholm, Sweden, participated. Interview questions on media were asked as part of a more extensive questionnaire. Informants’ responses were analysed qualitatively. The results indicate that doctors to some extent used media as a source of information, due to limited access to knowledge about the virus. Results further suggest that media coverage triggered fear of infection, caused worry and job strain. The doctors percieved that the media coverage on Covid-19 affected patient-seeking behaviour as well as the doctor-patient relationship. The findings can be relevant in preparation for future pandemics and considered in development of policy for media and emergency departments.

## Background

The Covid-19 pandemic burdened healthcare systems globally and created strained job situations in many healthcare settings, including emergency departments (EDs). Consequently, the well-being of doctors was affected substantially following increased distress, moral distress, anxiety, depression, and stress.^1-5^ The emotional distress experienced by healthcare workers during the pandemic was further negatively affected by the media coverage, as confusion and conspiracy theories circulated in the news and traditional media channels online.^6^ The need for information increased drastically during the pandemic, as it normally does during a crisis, generating an intensified consumption of media.^7^ Research has shown that the number of searches for information on the internet regarding Covid-19 increased as the infection spread during the pandemic.^8^ Over time, the spread of information about the Covid-19 virus escalated to such degree that it became problematic and was considered an ‘Infodemic’ by the World Health Organization, due to extensive problems with misinformation.^9^ For example, rumours and misinformation directly resulted in more than 800 deaths among people who drank bleach as they wrongly believed it was a cure for the virus.^10^ Misinformation has further been linked to targeted attacks on healthcare workers and worsened working conditions during previous infectious outbreaks such as the Ebola outbreak in 2014, as well as negative social consequences, health outcomes and spread of infection during the Covid-19 pandemic.^11-14^

Media coverage as part of the global infodemic during Covid-19 highly affected the public’s trust in healthcare workers and the healthcare system.^15^ According to researchers in journalism, at the beginning of the pandemic, Swedish traditional media primarily reported on the development of the spread of the coronavirus and information on the latest recommendations on how to avoid being infected.^16,17^ Yet, an analysis of Swedish media of year 2020 shows that media coverage was generally alarmistic, consistent with the public view; 67% perceived it as *very* or *somewhat* alarmistic.^17^ Previous research from the US has shown that the tone and narrative of media reports, as well as the political angle of the news channel, influenced the spread of infection. Citizens who consumed news from the more right-angled TV channel Fox News engaged in fewer preventative and more risky behaviours related to Covid-19, compared to those who consumed news from the more left-leaning channel CNN.^18^

The general view of media coverage, combined with the effects of media coverage on infection rates and pandemic outcomes, further raises the question of how media coverage has affected the well-being of doctors at emergency departments, a group whose health was highly affected by the pandemic.^4^ There is limited research on how traditional media coverage was perceived by and affected healthcare staff. One of few studies on the topic has been conducted in Sweden, showing that media coverage during the Covid-19 pandemic affected staff in care homes negatively, as the informants experienced that the media had provided an inadequate picture with incomplete and negative reporting on the role of care homes. This negatively influenced staff and their workplace in the form of feelings of sadness and shame.^19^ Yet, no study in Sweden has examined how the media coverage on Covid-19 affected frontline healthcare staff despite their daily contact with Covid-19 patients. To learn from the Covid-19 pandemic and prepare for future pandemics, it is of interest to study the role of media, including how media influenced medical staff. Therefore, this study has explored how doctors in emergency departments in Stockholm perceived the media coverage in Sweden during the Covid-19 pandemic.

## Study Aim

To explore medical doctors’ perceptions of media coverage during the Covid-19 pandemic.

## Methods

### Study design and setting

The study was performed using semi-structured interviews with medical doctors working at two hospital emergency departments (ED) in Stockholm, the capital of Sweden. Stockholm was selected as it was particularly negatively affected by the Covid-19 pandemic.^20^ The EDs were purposively sampled to allow for one hospital in central Stockholm and one outside central Stockholm.

### Participants

The participants consisted of medical doctors at the EDs of the two hospitals. Doctors at the two EDs received information about the study by their managers via posters and emails. Those who expressed an interest in participating received written information about the study and their rights to withdraw, as well as phone number and email address to project leader CB. They were then invited to an interview at a date and time convenient to them. The participants were invited to ask questions about their participation and the study during the arrangements and before the start of the interview. The study was undertaken as a graduate student’s degree project. Studies undertaken by students do not require ethical approval according to Swedish law on ethical review (2003:460 2§), still the study was conducted in accordance with national ethical standards including participants’ rights to information, withdrawal and anonymity. All informants gave formal consent to participate in the study in writing or verbally using the audio-recorder before the interview started.

### Interview questions

The interview guide contained questions on the informants’ experience of moral distress during and prior to the Covid-19 pandemic, and their ways of dealing with morally distressful events. Two questions were asked about how the participant experienced the media coverage during the Covid-19 pandemic:

1. How did the media coverage on Covid-19 and related mortality affect your work?
2. Did the intense media reporting affect the way you acted in situations where you experienced moral distress?

If necessary, follow-up questions were asked for clarification. The interview questions were inspired from a previous study conducted in Stockholm on the experience of media coverage during the Covid-19 pandemic.^19^ A pilot interview was conducted to confirm that the order of the questions and that the wording seemed logical and understandable.

### Procedure of the interviews

In total, 12 medical doctors were interviewed as saturation was reached with that sample size. See table 1 for demographics. There were no dropouts. The interviews (6 interviews at each ED, one per person) were undertaken between 15th December 2021 to 31st January 2022. Due to Covid-19 restrictions or work practicalities, 3 interviews were conducted digitally on Zoom. Two of these were at the hospital outside central Stockholm and one at the hospital in central Stockholm. The remaining 9 interviews were conducted at the hospitals where the participant worked. The interviews lasted between 42 and 95 minutes and were digitally audio-recorded and transcribed verbatim by CB. Field notes were taken following each interview. Identifiable information in the transcripts was de-identified. Following the transcription, the informants were given the opportunity to read the transcripts. The preliminary results were further shared with the informants who were invited to read and comment.

**Table 1.** Information about the informants.

*Informants*	*Age*	*Gender*	*Worked since*	*Position*
*1*	*50-55*	*Male*	*2014*	*Specialist*
*2*	*30-35*	*Male*	*2018*	*Resident*
*3*	*45-50*	*Male*	*2016*	*Last year resident*
*4*	*35-40*	*Female*	*2016*	*Last year resident*
*5*	*35-40*	*Male*	*2018*	*Resident*
*6*	*25-30*	*Male*	*2019*	*Resident*
*7*	*30-35*	*Female*	*2018*	*Licenced doctor*
*8*	*25-30*	*Male*	*2016*	*Resident*
*9*	*30-35*	*Male*	*2019*	*Resident*
*10*	*50-55*	*Male*	*2019*	*GP-specialist*
*11*	*25-30*	*Female*	*2019*	*Resident*
*12*	*30-35*	*Female*	*2012*	*Specialist*

CB (female), who is a medical student with experience in interview techniques, conducted the interviews.

### Data analysis

**Figure 1.**
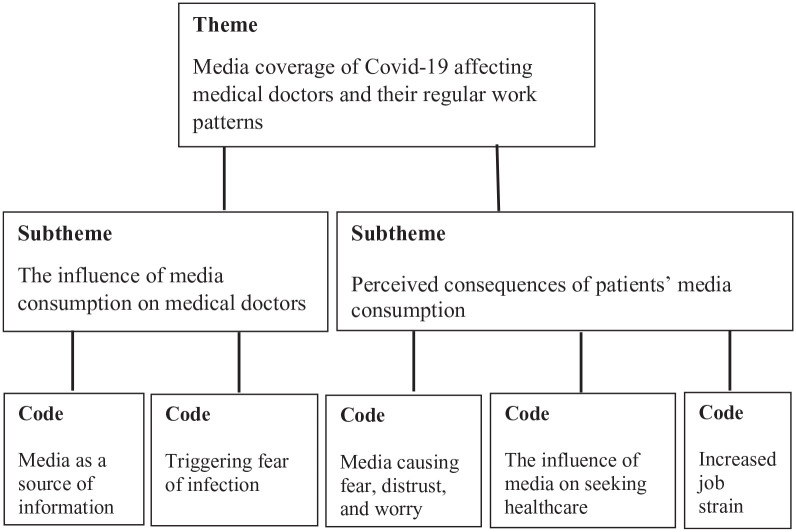
Themes, subthemes and codes identified in the data.

The data collected through the interviews were analysed through thematic analysis as outlined by Braun and Clarke.^21^ Interview transcripts were read by 3 researchers (CB, AL and JA). The transcripts were later discussed and analysed, and tentative codes were identified by CB and reviewed by AL and JA. The preliminary codes were collected in MS Word and were refined through discussions between the 3 researchers. The data set, organised by codes, was divided into preliminary themes and subthemes, further re-revised and agreed upon by CB, AL and JA (Figure 1). During the translation of codes, subthemes, themes and quotes from Swedish to English, synonyms and choice of words were continuously discussed to make translations as accurate as possible.

## Results

### Influences of doctors’ media consumption

This subtheme consists of 2 codes; ‘Media as a source of information’ and ‘Triggering fear of infection’.

#### Media as a source of information

Most informants reported that the media had filled a purpose as a source of information about the pandemic globally and in Sweden. Some thought that media reported in an objective manner about the situation in hospitals, at least at the beginning of the pandemic.

*‘At the beginning I felt it was good, for example the press conferences. . .’ Informant 12*

*‘. . .the public was made aware of what it was like to care for Covid-infected patients, and how sick they were’. Informant 3*

Some informants reported that the media gave necessary information about the strained situation in healthcare and highlighted the lack of personal protective equipment (PPE) for healthcare staff. One informant had used media as a tool to spread information about the situation in the ED. Some informants reported that the media coverage had affected them negatively as they considered it skewed and non-objective.

*‘. . .they [the media] have a great responsibility and an extreme amount of power, but I don’t think they’ve reported from a societal perspective, or done enough to help society, rather the opposite’. Informant 8*

Some informants thought that there had been insufficient media coverage regarding the importance of vaccination in favour of vaccine side effects, yet without highlighting the dangers accompanying a Covid-infection. Some informants expressed that media coverage exaggerated the pandemic situation. One informant reflected on their own view on unvaccinated patients which they considered had become more negative, due to stigmatization of unvaccinated people in the media. Informants also criticized media for mainly focusing on unique cases, rather than providing more objective information and facts about the disease. Most informants further reported that the media had an obsessive focus on how many had died from Covid-19, at the cost of more important aspects of the pandemic.

*‘There were daily reports of the number of deaths and the number of infected people, but they never reported on patients who were fine and sent home, and I think that’s important. The media scared everyone, and there were lots of rumours going around all the time. It felt like there was never any correct information, and that society just received the worst picture of the pandemic all the time’. Informant 8*

Most informants thought that the media coverage had not affected their actions in morally distressing situations such as having to prioritize which patients to treat or admit. One informant reported that media had affected their medical assessment in some cases since they had developed a false sense of the prevalence of blood clots in young patients.

*‘I ordered an extra X-ray just to see that there weren’t any clots in this case, because from media I had got a picture of it [blood clots] to be common, since media got to us faster than research findings. . .’ Informant 8*

#### Triggering fear of infection

Some informants reported that the media had a negative influence on the care situation and society as it triggered worry among themselves including fear of getting ill.

*‘Initially, I was a bit afraid of getting ill’. Informant 1*

Some reported that the information provided by the media on the pandemic globally, including countries where the spread had rapidly escalated, created worry regarding the spread of the pandemic in Sweden, and how it would affect the EDs.

*‘In the beginning of the pandemic, there was a bit of a “doomsday” feeling. . .’ Informant 11*

### Perceived consequences of patients’ media consumption

There are 3 codes under this subtheme: ‘Media causing fear, distrust and worry’ ‘The influence of media on seeking healthcare’ and ‘Increased job strain’.

#### Media causing fear, distrust, and worry

Some informants reported that the general worry in society created by the media coverage of the pandemic caused distrust among patients directed towards healthcare workers. Examples given included news items on vaccine side effects, resulting in patients questioning the doctors’ decisions regarding treatment and admission.

*‘. . .they [patients] question our medical judgement because they’ve read something in a forum or magazine’. Informant 6*

#### The influence of media on seeking healthcare

One informant mentioned that the frequent media reporting on vaccine side-effects created a new category of patients who sought emergency healthcare because they had been vaccinated and were worried. Some informants expressed feelings of annoyance directed towards patients and the media.

*‘Following all the media posts on vaccine side effects, especially Astra Zeneca, there was a time – a lot less now though – when a lot of patients sought help for unspecific symptoms as they had questions about side effects, which caused an increased workload. . .’ Informant 6*

Some informants further reported that the media had affected the way patients seek healthcare in general. For example, some reported that fewer patients sought healthcare during certain periods of the pandemic. One informant reported on a situation where a young patient infected with Covid-19 arrived at the ED with cardiac arrest, whose life could not be saved. Later, the informant was told that the patient had chosen not to seek healthcare earlier due to intense media coverage on the burdened healthcare sector and fear of getting infected in the ED.

*‘What you noticed during the first wave – and I think that was caused by the media attention – was that people didn’t come to the ED as much [as before the pandemic]’ Informant 3*

Some informants reported that patients consuming media were better informed and had a greater understanding of the pressured situation in the hospitals with long waiting hours.

#### Increased job strain

Most informants reported that the media coverage’s influence on patients had increased their workload. One informant also reported that what they themselves had read and heard about in the media occasionally influenced what they told their patients and to what extent they had to explain their treatment decisions.

*‘It [the media] created worry, which spread in society, and was brought here by the patients. And we, the healthcare workers, had to respond to all the worry, all the anxiety and all the fear that the media had triggered’. Informant 8*

*‘. . .we got an increased workload and the patients did not have the same trust in us doctors when we made some clinical judgements, because they had read or seen something else’. Informant 6*

Some informants reported that their work had not been affected by the media coverage on Covid-19.

## Discussion

The results indicate that the interviewed doctors perceived that media coverage during the Covid-19 pandemic caused worry and affected their work burden. The doctors’ work burden increased as patients questioned and doubted treatment decisions. The informants reported that worry caused some patients to seek more healthcare as they feared they had a severe Covid-19 infection or suffered serious side effects of the vaccine. Media was reported to have a negative influende on the work situation through increasing distrust from patients and workload for the doctors, fear of infection and work-related stress. In contrast, some experienced that there were people who sought less healthcare as they were afraid of getting infected or did not want to burden the healthcare system. As opposed to the negative views on the media coverage, the informants also considered the media to provide necessary information about the situation, which is consistent with perspectives reported in previous research.^22^ Some informants further thought that the media coverage contributed to patients being informed about the pandemic and the strained situation in the EDs. Indeed, the most reported aspects of the pandemic in Swedish media referred to restrictions, the spread of infection, and vaccines.^17^

Consequently, media reports during the Covid-19 pandemic contained both positive and negative elements. The informants’ experience of patients questioning the doctors’ medical decisions to a greater extent during the pandemic adds to a new trend in distrust and insecurity in doctors and the healthcare system, shown to affect the doctor-patient relationship.^23-26^ The participating doctors experienced that this was caused by the media coverage and rumours regarding vaccine side effects, and alarmist reports amplifying the perceived proportion of severely ill patients. The spread of misinformation and rumours during the ‘Infodemic’, and the amount of research published without being peer-reviewed could be considered as an explanation for this insecurity.^27,28^ Swedish citizens however generally trust health services^29^ and its staff: a survey conducted during the pandemic has shown that 90% of Swedes trusted information in the media presented by healthcare workers and scientists, but only 20% trusted information presented by journalists.^17^ According to Habermas theory of communication, the public sphere is a discursive arena where information and arguments are tested against each other through communicative action, enabling the better argument to win rather than the position of the speaker.^30^ The public’s trust in scientists and healthcare workers during the pandemic could refer to more trustworthy informative arguments from scientists and healthcare workers, in combination with this group being more trustworthy in the pandemic context. Similar to Habermas’ ideas that ‘social pathologies can be understood as forms of manifestation of systematically distorted communication . . .’,^31^ the infodemic and its effect on healthcare workers might reflect how the public sphere intervened with crisis management.

The positive role of media reports during the pandemic included disseminating valuable information about the pandemic. In this study, one informant reported on how they had used the media to spread information about the insufficient staffing in their emergency department and its consequences, such as delayed treatment and long waiting hours. Such information is likely to receive attention among the general public as it puts patient safety at risk. Using the media to generate attention about poor work conditions in the care sector has previously been a successful strategy to improve the work situation for midwives.^32^ Poor working conditions in the EDs has further been reported in an employee survey. In the summer 2022, the Swedish Medical Association presented the results of their workplace environment survey showing that medical doctors are least satisfied with the workplace environment in emergency departments.^33^ Whilst this study adds to existing findings on challenging work situations in the ED by showing that the media reporting caused worry among staff, increased workload and job strain, further research is needed to evaluate the workplace health in EDs. Surely the increase in workload and job strain are particularly problematic during a pandemic when there is a shortage of staff due to illness. Still, the pandemic highlighted challenges in the work environment at EDs which need to be considered in the development of policies for the ED setting and preparation to manage the pressure on the ED in future pandemics.

Limitations to this study include that the interview questions were asked as part of a questionnaire focusing on the experiences of moral distress during the pandemic. The different focus might have affected the informants’ way of considering the question and the relevance of their experience of media coverage, unrelated to moral distress. Further, only 2 questions about media were asked, providing limited data. If the interview had focused entirely on media coverage, more aspects and in-depth data on the media reporting would have been included. The questions were however followed up when needed, and data were collected until saturation was reached. Another limitation to the study is that it cannot be guaranteed that the informants were always referring to traditional media rather than social media during the interviews. Moreover, it is unknown to what extent informants were influenced by their own media consumption versus the media consumption of other people who they trust and talk to, including people who confirm their thoughts. Further, qualitative studies contain some inherent limitations. Interviewer, as well as interviewee biases, can have an impact on both the data and the analysis, influencing the results. These possible issues were mitigated through the analysis process being conducted through several steps and both individually and jointly. The dataset was based on a small sample size, which means it does not represent the views of all doctors in Sweden, particularly as the sample set was only collected from one Swedish region. However, the different sizes of the hospitals may have provided different valuable perspectives, relevant for adapting policies to different types of hospitals.

## Conclusions

In conclusion, medical doctors from the EDs in Stockholm who participated in the study perceived that media coverageduring the Covid-19 pandemic resulted in patients being somewhat ‘desinformed’, which to some extent changed people’s ED-seeking behaviour. The media coverage further caused worry among both doctors and patients. Initially, doctors worried about getting infected. Patients worried about side-effects of the vaccines and potential consequences of infection, resulting in increased workload, in the already burdened situation. Furthermore, the doctor-patient relationship was thought to be affected by distrust as patients questioned treatment decisions to a greater extent. As doctors were affected by the media coverage during the pandemic, possibly negatively influencing their well-being, there are implications for developing policy in the ED and media, and in preparation for future pandemics. Examples include media directly communicating with hospitals before spreading information on the hospital situation, and media providing clear instructions to the public on when to visit the ED and with what approach. Further, EDs could provide their staff with tools to address media-specific questions and worry from patients.
